# Salivary parameters and periodontal inflammation in obstructive sleep apnoea patients

**DOI:** 10.1038/s41598-022-23957-5

**Published:** 2022-11-12

**Authors:** Mia Tranfić Duplančić, Renata Pecotić, Linda Lušić Kalcina, Ivana Pavlinac Dodig, Maja Valić, Marija Roguljić, Dunja Rogić, Ivana Lapić, Katarina Grdiša, Kristina Peroš, Zoran Đogaš

**Affiliations:** 1 School of Medicine, University of Split, 21000 Split, Croatia; 2 University Hospital Center Zagreb, 10000 Zagreb, Croatia; 3 Department of Pharmacology, School of Dental Medicine, University of Zagreb, Šalata 11, Zagreb, Croatia

**Keywords:** Sleep disorders, Diagnostic markers, Periodontitis

## Abstract

The aim of this cross-sectional study was to objectively assess the salivary flow rate and composition and periodontal inflammation in obstructive sleep apnoea (OSA) patients. The subjects, who underwent whole-night polysomnography or polygraphy, were referred for saliva sampling and periodontal examination. According to the severity of OSA based on the Apnoea Hypopnea Index (AHI) value, the subjects were classified into groups: no OSA (AHI < 5; N = 17), mild to moderate OSA (AHI 5–29.9; N = 109), and severe OSA (AHI > 30; N = 79). Salivary flow rate, pH, salivary electrolytes, and cortisol were measured from collected saliva samples. Periodontal examination included assessment of the number of teeth, dental plaque, bleeding on probing and periodontal measurements: gingival recession, probing pocket depth, clinical attachment level (CAL) and periodontal inflamed surface area (PISA) score. There were no significant differences in salivary flow rate, salivary pH, salivary electrolyte concentrations or electrolyte ratios among the groups classified according to the severity of OSA. However, subjects without OSA had higher salivary cortisol concentrations than OSA groups (p < 0.001). Increased plaque scores were associated with a higher AHI (r = 0.26; p = 0.003). According to the salivary flow rate, subjects with hyposalivation and reduced salivation had higher concentrations of salivary electrolytes and lower salivary pH than subjects with normal salivation. Subjects with hyposalivation had an increased Mg/PO_4_ ratio (p < 0.001) and a reduced Ca/Mg ratio (p < 0.001). Furthermore, subjects with severe OSA tended to have higher CALs and plaque volumes. In conclusion, under pathological conditions, such as OSA, multiple interactions might impact salivary flow and electrolyte composition. Complex interrelationships might affect the integrity of oral health, especially considering OSA severity, inflammation, concomitant diseases and medications.

## Introduction

Saliva is responsible for the protection of intraoral structures, maintaining healthy hard and soft oral tissues while taking part in digestion and articulation of speech. Because of the buffer systems, saliva helps to maintain an acceptable pH range, whereas its components can provide information about caries formation or inflammation of the oral cavity^1–3^.

Salivary flow rate can be measured by objective examination techniques as previously described by Navazesh and Kumar^4^, providing insight into oral and general health^2^. More precisely, salivary flow rate may be modulated by many local factors affecting the oral cavity, such as mouth breathing and smoking, as well as therapies and systemic diseases, such as diabetes mellitus^5^. Waking up with a dry mouth is often reported by patients with obstructive sleep apnoea (OSA), a sleep-related breathing disorder characterized by sleep fragmentation and recurrent collapses of the upper airway during sleep^6^. Furthermore, it has been shown that hyposalivation is more frequent in OSA patients than in healthy subjects^7^. However, these findings were based on reports of the subjective feeling of dry mouth in OSA patients. Makeeva et al., using both subjective evaluation and objective measurement of salivary flow rate, found that patients with severe OSA indeed had a reduction in their salivary flow rate^8^. A reduced salivary flow rate leads to changes in saliva composition that could increase plaque accumulation and the risk for caries, mucosal and gingival infection, and inflammation^9^.

One of the most common immune-inflammatory diseases of the oral cavity is periodontitis^10^. Periodontal clinical parameters such as the clinical attachment level (CAL) are important to evaluate the severity of periodontitis, whereas bleeding on probing (BoP), probing pocket depth (PPD) and periodontal inflammation surface area (PISA) provide information on the severity of periodontal inflammation^11^. However, in addition to the clinical estimation, periodontal inflammation could also be determined by biochemical markers^12^. The determination of saliva composition and salivary flow rate could be considered an option for extended diagnostics of local and systemic inflammation^12–14^. More specifically, increased levels of salivary calcium and phosphate, as well as an imbalance in the Mg/Ca ratio and alterations in the salivary flow rate, may be associated with periodontitis^15,16^.

Evidence from the literature indicates that saliva should be considered a useful and noninvasive diagnostic tool, but it has still been underutilized for routine diagnosis of respiratory diseases^17^. Furthermore, recent systematic reviews and meta-analyses^18,19^ confirmed the association of OSA and periodontitis. One of the possible explanations might be disorders of the immune system, since sleep fragmentation, as one of the key features of OSA, has a deep impact on immunity and as such might contribute to periodontal inflammation^20^. An assessment of saliva composition, flow rate and periodontal inflammation may facilitate diagnosis and monitoring of the progression of oral diseases that might be enhanced in OSA patients. Thus, the aim of this study was to objectively assess the salivary flow rate and composition, along with periodontal inflammation, in OSA patients.

## Results

Out of 220 recruited patients, 209 (135 men and 70 women) agreed to participate in this study, leading to a response rate of 95%.

The demographic characteristics of the subjects, as well as their subjective assessment of dry mouth and oral hygiene habits, are shown in Table 1. Out of 205 subjects, 17 (35.3% male, 64.7% female) did not have OSA (Apnoea Hypopnea Index [AHI] score < 5), 109 (62.4% male, 37.6% female) subjects were diagnosed with mild to moderate OSA (AHI 5–29.9) and 79 (77.2% male, 22.8% female) had severe OSA (AHI > 30) (Table 1).

**Table 1:** Baseline characteristics of the subjects and data collected by the questionnaire according to OSA severity.

	No OSA N = 17	Mild to moderate OSA N = 109	Severe OSA N = 79	p
Age (years)	37 (31–51) ^1^	58 (50–67) ^2^	61 (51–68) ^2^	< 0.001
BMI (kg/m^2^)	25.1 (23.5–27.5) ^1^	28.7 (25.6–31.9) ^1^	31.4 (28.35–35.5) ^1^	< 0.001
AHI (events/h)	1.3 (1–3.4) ^1^	11.9 (8–17) ^1^	40 (33–58.7) ^1^	< 0.001
**Sex**				
Male	6 (35.3)	68 (62.4)	61 (77.2)	0.002
Female	11 (64.7)	41 (37.6)	18 (22.8)	
**Hypertension**				
Yes	3 (17.6)	63 (57.8)	50 (63.3)	0.002
No	14 (82.4)	46 (42.2)	29 (36.7)	
**DM II**				
Yes	1 (5.9)	14 (12.8)	17 (21.5)	0.139
No	16 (94.1)	95 (87.2)	62 (78.5)	
**Smoking**				
Yes	4 (23.5)	30 (27.5)	14 (17.7)	0.293
No	13 (76.5)	79 (72.5)	65 (82.3)	
**Dry mouth upon awakening**				
Never	3 (17.6)	16 (14.7)	7 (8.9)	0.233
Rarely	4 (23.5)	22 (20.2)	11 (13.9)	
Sometimes	4 (23.5)	29 (26.6)	15 (19.0)	
Often	5 (29.4)	22 (20.2)	29 (36.7)	
Almost always	1 (5.9)	20 (18.3)	17 (21.5)	
**Do you often feel that your mouth is dry?**				
Yes	7 (41.2)	41 (37.6)	30 (38)	0.961
No	10 (58.8)	68 (62.4)	49 (62)	
**Is your mouth dry while eating?**				
Yes	0 (0)	3 (2.8)	1 (1.3)	0.638
No	17 (100)	106 (97.2)	78 (98.7)	
**Do you have difficulty swallowing dry food?**				
Yes	1 (5.9)	39 (35.8)	15 (19)	0.005
No	16 (94.1)	70 (64.2)	64 (81)	
**Do you have to drink constantly to make it easier to swallow dry food?**				
Yes	3 (17.6)	22 (20.2)	13 (16.5)	0.806
No	14 (82.4)	87 (79.8)	66 (83.5)	
**Do you have enough saliva in our mouth?**				
Yes	16 (94.1)	97 (89)	72 (92.3)	0.654
No	1 (5.9)	12 (11)	6 (7.7)	
**Dental visits (in a year)**				
Never	1 (5.9)	15 (13.8)	23 (29.1)	0.020
Less than once	1 (5.9)	21 (19.3)	11 (13.9)	
At least once	15 (88.2)	73 (67)	45 (57)	
**Tooth brushing (in a day)**				
Never	0 (0)	8 (7.3)	9 (11.4)	0.051
Once	2 (11.8)	30 (27.5)	26 (32.9)	
Two times	11 (64.7)	65 (59.6)	39 (49.4)	
More than two times	4 (23.5)	6 (5.5)	5 (6.3)	
**Use of toothpaste that contains fluoride**				
Yes	15 (88.2)	102 (94.4)	71 (91)	0.524
No	2 (11.8)	6 (5.6)	7 (9)	
**Dental floss**				
Regularly	2 (11.8)	15 (13.8)	1 (1.3)	0.004
Sometimes	6 (35.3)	29 (26.6)	13 (16.5)	
No	9 (52.9)	65 (59.6)	65 (82.3)	
**Mouthwash (antiseptic)**				
Yes	10 (58.8)	32 (29.4)	13 (16.5)	0.001
No	7 (41.2)	77 (70.6)	66 (83.5)	

Data are presented as the medians with interquartile ranges. The Kruskal‒Wallis test was used for comparisons among groups. Categorical data are presented as frequencies (percentages). The chi-square test or Fisher’s exact test was used for comparisons among groups.

^1^Post hoc difference to both other groups following the Mann‒Whitney U test p < 0.05.

^2^Post hoc difference only to the no OSA group following the Mann‒Whitney U test p < 0.05.

Among subjects with no OSA, the median age was 37 (31–51) years, and the average body mass index (BMI) was 25.1 kg/m^2^. Among subjects with mild to moderate OSA, the median age was 58 (50–67) years, and the average BMI was 28.7 kg/m^2^, whereas among subjects with severe OSA, the median age was 61 (51–68) years, and the average BMI was 31.4 kg/m^2^.

Subjects with severe OSA were older (p < 0.001) and had a higher body mass index (p < 0.001) than those in the two other groups. Regarding concomitant diseases, 17.6% of subjects with no OSA had hypertension, whereas 57.8% of subjects with mild to moderate OSA and 63.3% of subjects with severe OSA had hypertension (p = 0.002). There was no significant difference in the frequency of diabetes mellitus type 2 among the studied groups (5.9% in the no OSA, 12.8% in the mild to moderate OSA, and 21.5% in the severe OSA groups; p = 0.139) (Table 1). The investigated groups differed significantly only in the use of antihypertensives (p = 0.010), which is in accordance with the data collected on systemic diseases (Supplementary Table 1).

### Saliva composition

There was no statistically significant difference between the groups in terms of subjective dry mouth upon awakening (p = 0.233) and during the daytime. However, subjects with mild to moderate OSA most frequently reported difficulties swallowing dry food (p < 0.005). Although the subjects with no OSA did not report dry mouth while eating, there was no statistically significant difference between the groups (Table 1).

The subjects without OSA and those with mild to moderate OSA had more frequent dental check-ups during the year (p = 0.020) and had better oral hygiene habits, such as dental flossing (p = 0.004) and using mouthwash (p = 0.001), than the subjects with severe OSA (Table 1).

The salivary flow rate and salivary parameters in the investigated groups are summarized in Table 2.

**Table 2:** Salivary and periodontal parameters according to OSA severity.

	No OSA N = 17	Mild to moderate OSA N = 109	Severe OSA N = 79	p
**Salivary parameters**				
Salivary flow (ml/min)	0.244 (0.15–0.39)	0.23 (0.15–0.36)	0.22 (0.14–0.41)	0.918
Salivary pH	6.58 (6.47–6.74)	6.59 (6.36–6.84)	6.64 (6.33–7)	0.286
Salivary calcium (mmol/L)	0.61 (0.47–0.8)	0.51 (0.38–0.69)	0.52 (0.39–0.69)	0.590
Salivary phosphate (mmol/L)	6.39 (4.31–8.24)	6.19 (5.04–7.49)	6.43 (4.64–8.85)	0.801
Salivary magnesium (mmol/L)	0.2 (0.15–0.31)	0.19 (0.14–0.27)	0.19 (0.14–0.27)	0.874
Ca/Mg	2.67 (2.15–3.03)	2.59 (1.95–3.8)	2.62 (1.88–4.12)	0.991
Ca/PO_4_	0.09 (0.08–0.11)	0.09 (0.06–0.12)	0.08 (0.06–0.12)	0.842
Mg/PO_4_	0.03 (0.03–0.04)	0.03 (0.02–0.04)	0.03 (0.02–0.04)	0.678
Salivary cortisol (ng/mL)	6.25 (5.156–8.13) ^1^	4.64 (3.032–5.75) ^2^	4.57 (3.11–6.06) ^2^	0.011

	N = 11	N = 87	N = 59	
**Periodontal status**				
Number of teeth	28 (25.5–28) ^1^	23 (17–27) ^2^	22 (15.5–25.5) ^2^	0.019
Plaque (%)	38 (29.5–51) ^1^	59 (46–78) ^1^	73 (59.3–94) ^1^	0.001
BoP (%)	20 (6–45)	21 (10–32)	20 (12.3–36)	0.957
CAL (mm)	2.3 (2–2.7)	2.6 (2.2–3)	2.7 (2.1–3.4)	0.175
PISA score (mm^2^)	173.7 (67.1–546.4)	218.3 (89.5–368.8)	179.7 (86.65–344.94)	0.953
**Stages of periodontitis**				
No periodontitis	3 (27.3)	7 (9.1)	7 (13)	0.095
Periodontitis stages 1 and 2	6 (54.5)	33 (42.9)	20 (37)	
Periodontitis stages 3 and 4	2 (18.2)	37 (48.1)	27 (50)	

Data are presented as the medians with interquartile ranges. The Kruskal‒Wallis test was calculated for group comparisons.

^1^Post hoc difference to both other groups following the Mann‒Whitney U test p < 0.05.

^2^Post hoc difference only to the no OSA group following the Mann‒Whitney U test p < 0.05.

There were no significant differences in salivary flow rate, salivary pH, or salivary calcium, phosphate, or magnesium concentrations or their ratios among the groups. However, subjects without OSA had higher salivary cortisol concentrations than those who had OSA (p = 0.011).

### Periodontal assessment

Regarding periodontal status, the subjects with severe OSA had fewer teeth than subjects without OSA (p = 0.019). Furthermore, subjects with OSA had more plaque than those without OSA (p < 0.001). There was no significant difference in BoP, CAL, PISA score or stage of periodontitis among the groups (Table 2).

Increased plaque scores were associated with a higher AHI (r = 0.26; p = 0.003) (Supplementary Table 2).

### Salivary flow rate

Regarding the salivary flow rate, the following categories were defined: hyposalivation (flow rate less than 0.1 mL/min, N = 25), reduced salivation (flow rate 0.1–0.3 mL/min, N = 97) and normal salivation (flow rate > 0.3 mL/min, N = 66) (Table 3).

**Table 3:** Salivary and periodontal parameters according to salivary flow rate.

	Hyposalivation (flow rate less than 0.1 mL/min) N = 25	Reduced salivation (flow rate 0.1–0.3 mL/min) N = 97	Normal salvation (flow rate more than 0.3 mL/min) N = 66	p
Age (years)	61 (53–70)	60 (52–68)	58.5 (49–66.75)	0.487
**Sex**				
Male	14 (56%)	69 (71.1%)	52 (78.8%)	0.095
Female	11 (44%)	28 (28.9%)	14 (21.2%)	
BMI (kg/m^2^)	31.6 (27.1–36.8)	29.7 (26.2–32.9)	30.1 (27.82–32.92)	0.283
AHI (events/h)	26.8 (10.9–46.8)	20.1 (11–34.9)	19.5 (10.52–36.5)	0.672
**Salivary parameters**				
Salivary pH	6.36 (6.06–6.78) ^3^	6.53 (6.3–6.76) ^3^	6.89 (6.62–7.16) ^1,2^	< 0.001
Salivary calcium (mmol/L)	0.67 (0.47–0.94) ^3^	0.56 (0.41–0.71) ^3^	0.42 (0.34–0.57) ^1,2^	< 0.001
Salivary phosphate (mmol/L)	8.38 (4.91–11.27) ^3^	6.93 (5.48–8.77) ^3^	5.35 (4.46–6.43) ^1,2^	< 0.001
Salivary magnesium (mmol/L)	0.42 (0.34–0.6) ^2,3^	0.2 (0.16–0.25) ^1,3^	0.15 (0.1–0.19) ^1,2^	< 0.001
Ca/Mg	1.8 (1.35–2.48) ^2,3^	2.65 (1.92–3.78) ^1^	3 (2.15–4.67) ^1^	< 0.001
Ca/PO_4_	0.09 (0.06–0.12)	0.09 (0.06–0.12)	0.09 (0.06–0.12)	0.842
Mg/PO_4_	0.05 (0.03–0.06) ^2,3^	0.03 (0.02–0.04) ^1^	0.03 (0.02–0.03) ^1^	< 0.001
Salivary cortisol (ng/mL)	5.44 (4.59–6.58) ^3^	5.02 (3.59–6.13) ^3^	3.88 (1.7–5.56) ^1,2^	0.003

	N = 17	N = 75	N = 50	
**Periodontal status**				
Number of teeth	20 (16–24)	21 (16–27)	24 (18–27)	0.269
Plaque (%)	59 (52–100)	63 (49.5–80.5)	67 (53.5–80.5)	0.894
BoP (%)	27 (14–29)	22 (12.5–37)	17 (7.5–28.5)	0.234
CAL (mm)	2.5 (2.3–3)	2.7 (2.3–3)	2.6 (1.95–3.05)	0.573
PISA score (mm^2^)	240.2 (111.1–349.8)	238.29 (94.22–373.1)	147.6 (74.6–331.4)	0.245

Data are presented as the medians with interquartile ranges. The Kruskal‒Wallis test was used for group comparisons.

^1^Post hoc difference significant to < 0.1 per group following the Mann‒Whitney U test.

^2^Post hoc difference significant to 0.1–0.3 per group following Mann‒Whitney U test.

^3^Post hoc difference significant to > 0.3 per group following Mann‒Whitney U test.

There was no significant difference in sex, age or body mass index between the groups.

Subjects with hyposalivation and reduced salivation had significantly lower pH levels (p < 0.001) and higher salivary calcium (p < 0.001), salivary phosphate (p < 0.001), and salivary cortisol concentrations (p = 0.003) than subjects with normal salivation (Table 3).

The highest concentrations of salivary magnesium (p < 0.001) were found in subjects with hyposalivation. Consequently, subjects with hyposalivation had a significantly higher Mg/PO_4_ ratio (p < 0.001) and lower Ca/Mg ratio (p < 0.001) (Table 3).

There were no significant differences in periodontal status among the groups; however, subjects with hyposalivation tended to have higher PISA scores than those in the other two groups.

The linear regression analysis indicated an association between a higher salivary flow rate and male respondents (β = − 0.154, p = 0.016), lower salivary calcium concentrations (β = − 0.215, p = 0.027), salivary cortisol concentrations (β = − 0.176, p = 0.008), and a higher Ca/Mg ratio (β = 0.329, p = 0.001) (Table 4). When multivariable regression analysis was performed with 142 subjects who underwent periodontal examination, predictors recognized as relevant for the salivary flow rate were male sex and salivary phosphate concentration, indicating higher salivary flow with lower phosphate levels in saliva (Supplementary Table 3).

**Table 4:** Linear regression analysis according to salivary flow rate.

	B	SE	Beta	t	P	R^2^	p
						33%	< 0.001
AHI	0.001	0.009	0.007	0.104	0.917		
Age	− 0.020	0.014	− 0.096	− 1.405	0.162		
Sex (Male = 0)	− 0.886	0.363	− 0.154	− 2.442	0.016		
BMI	0.016	0.035	0.034	0.472	0.638		
Salivary calcium	− 1.833	0.820	− 0.215	− 2.236	0.027		
Salivary phosphate	− 0.145	0.091	− 0.155	− 1.586	0.115		
Salivary magnesium	− 3.238	2.972	− 0.143	− 1.089	0.278		
Ca/Mg	0.504	0.153	0.329	3.293	0.001		
Ca/PO_4_	− 0.643	0.851	− 0.048	− 0.756	0.450		
Mg/PO_4_	− 2.884	6.680	− 0.032	− 0.432	0.666		
Salivary cortisol	− 0.150	0.056	− 0.176	− 2.670	0.008		
Hypertension	0.600	0.385	0.109	1.558	0.121		
DM II	− 0.531	0.505	− 0.070	− 1.051	0.295		

Unstandardized beta (*B*); Standard error for the unstandardized beta (*SE*); Standardized beta (*β*); T-test statistic (*t*); Coefficient of determination (*R*^2^); Probability value (*p*).

## Discussion

In our study, subjects with hyposalivation or a reduced salivary flow rate had decreased values of salivary pH and a Ca/Mg ratio, whereas concentrations of calcium, magnesium, and phosphate and the Mg/PO_4_ ratio were higher compared to subjects with normal salivation. There was no statistically significant difference between the groups in terms of subjective dry mouth upon awakening and during the daytime. In addition, subjects with severe OSA had less frequent dental check-ups and poor interdental oral hygiene habits.

Dry mouth as a subjective sensation of oral dryness was very commonly reported among OSA patients. Previous studies among patients with OSA have used questionnaires to subjectively assess the presence of dry mouth and have reported that the prevalence of hyposalivation/xerostomia increased with the severity of OSA^6,7,21^. Similarly, self-reported dry mouth symptoms were found among patients at risk for OSA. However, when objective measurements of morning hyposalivation were performed using the Schirmer test, there was no correlation with the risk for OSA^22^.

Regarding the objective measurements of oral dryness, our study, along with that by Makeeva et al., collected saliva and measured salivary flow rate, as previously described by Navazesh^4^, among patients who underwent diagnostic polysomnography or polygraphy procedures in a sleep laboratory. In the study by Makeeva et al., hyposalivation was diagnosed in patients with severe OSA, and the salivary pH value was decreased. Possibly due to the larger sample size in our study, we found no differences in the salivary flow rate or pH value between the OSA groups. There is a reason to believe that changes in sympathetic outflow as seen in OSA patients might affect the regulation of the salivary flow rate^23,24^. When we used that approach and analysed the results regarding salivary flow rate, we found that subjects with hyposalivation had a lower pH than subjects with normal salivation. Although we found no significant difference in the severity of OSA concerning the salivary flow rate, subjects with hyposalivation had a tendency to have higher AHI values that require further investigation.

Some studies have shown that a decrease in the salivary flow rate can lead to an alteration of saliva composition in the context of different chronic diseases. Mata et al. showed a decreased salivary flow rate along with a higher salivary calcium concentration in subjects with diabetes mellitus^14^, whereas Ponciano et al. recorded a low concentration of salivary calcium and a high phosphate concentration along with hyposalivation in subjects with mucopolysaccharidosis^25^. In our study, salivary flow rate, salivary pH, and calcium, magnesium and phosphate concentrations did not differ according to the severity of OSA among subjects referred to a sleep laboratory. To the best of our knowledge, this is the first study that measured salivary electrolytes in patients referred to a sleep laboratory due to sleep-related breathing disorders. This study indicated no difference in salivary calcium, magnesium or phosphate concentrations associated with the severity of OSA. However, when we analysed the results with regard to salivary flow rate, we found that concentrations of electrolytes and salivary cortisol significantly increased with reduced salivary flow rate.

The salivary flow rate might be influenced by age and sex among healthy subjects^26^. More specifically, the salivary flow rate was greater in males than in females, and older age was associated with decreased unstimulated flow^27^. In our study, which was performed among predominantly OSA subjects, no significant difference was found in sex, age or body mass index between the groups according to the salivary flow rate. However, regression analysis revealed a significant effect of male sex on the increased salivary flow rate with concomitant diseases included in the analysis. In addition, concomitant diseases of OSA, such as hypertension and diabetes mellitus type 2, might be considered conditions that affect the salivary flow rate^28^. One might speculate that in OSA, an interplay of underlying pathophysiological mechanisms and specific phenotype markers could substantially modulate the salivary flow rate as well as the salivary composition.

The salivary cortisol concentration was higher in subjects without OSA than in those who were diagnosed with OSA. Plasma cortisol concentration could be used as an important marker for pathophysiological changes in sleep disorders^29^, and some studies yielded inconsistent results regarding plasma cortisol concentration and the severity of OSA^30^. In addition to plasma cortisol concentration, salivary cortisol concentration could be used in the assessment of OSA severity^31^, even though previous studies yielded inconsistent results regarding the association between salivary cortisol and the severity of OSA^32,33^.

The results from periodontal examination showed that subjects with OSA had more plaque and increased plaque scores that were associated with a higher AHI. A reduced salivary flow rate can lead to altered salivary pH and saliva composition, resulting in the accumulation of an increased number of microorganisms that form the oral biofilm, which is one of the most important aetiological factors for periodontitis^5,34,35^. Furthermore, in this study, an increased salivary calcium concentration was associated with the CAL, which might contribute to dental plaque formation. Salivary electrolytes may be involved in processes in periodontitis^16^, although some studies yielded inconsistent results^10,36,37^. Additionally, in this study, periodontal stages did not differ significantly with regard to OSA severity. However, subjects with severe OSA had a tendency to have higher CALs and plaque volumes than other subjects. Previous research showed that OSA severity is associated with severe forms of periodontitis^18,19,38^ and found that both diseases share similar risk factors that are associated with systemic inflammation^39–41^. Our study included a calculation of the PISA score as a novel method for determining periodontal inflammation in patients with sleep-related breathing disorders. The results of our study did not show a significant difference in the PISA score regarding OSA severity. However, subjects with hyposalivation had a tendency to have higher PISA scores than those in the other groups, indicating that hyposalivation and OSA might affect periodontal inflammation.

Among the limitations of this study, the cross-sectional design prevented conclusions on the causal association between saliva parameters and OSA. Furthermore, we lacked information on the precise wake time before sampling, which might have contributed to the concentrations measured. Although we tried to control for this effect by inviting subjects within a limited time frame in the morning, given the possible additional impact of the exact time between waking and saliva sampling, future studies are needed to more precisely elucidate the influence of saliva sampling time on salivary flow rate and composition.

In our study, subjects referred to a sleep laboratory with hyposalivation or a reduced salivary flow rate had altered saliva composition. In addition, higher CAL and plaque values were found in subjects with severe OSA. In conclusion, we argue that under pathological conditions, such as OSA, multiple interactions might impact salivary flow and electrolyte composition. Complex interrelationships might affect the integrity of oral health, especially considering OSA severity, inflammation, concomitant diseases and medications.

## Methods

### Subjects

**Figure 1:**
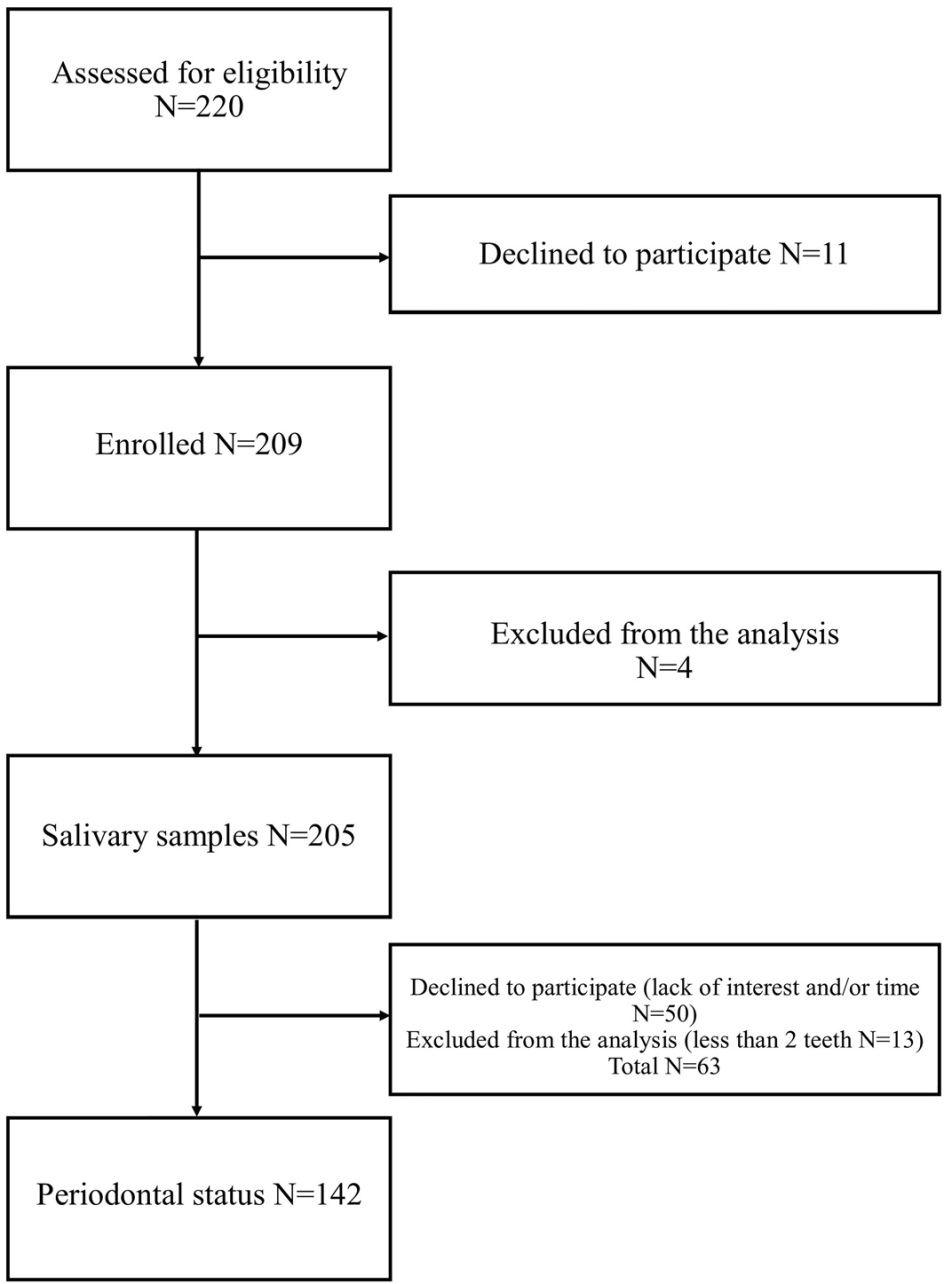
Flow diagram of the study subject enrolment process.

This cross-sectional study was conducted between November 2018 and October 2019. The subjects, who underwent whole-night polysomnography or polygraphy at the Split Sleep Medicine Centre (SMC), were invited to participate in this study. All 209 subjects included in this study signed an informed consent form for the use of personal data when accessing the survey and were provided with written information about the study. Subjects older than 18 years who underwent whole-night polysomnography (PSG, N = 51) or polygraphy (PG, N = 154) and refrained from consuming any food or beverage 2 h before saliva sample collection were included in this study (N = 205). Subjects younger than 18 years and those who did not adhere to the recommendation of fasting for 2 h prior to saliva sample collection were excluded from this study (N = 4) (Fig. 1). This study was approved by the Ethics Committee of the University of Split School of Medicine (Class: 003–08/14-03/0001, No: 2181–198-03-04-14-0027) and is in accordance with the Declaration of Helsinki. This study was conducted following the Strengthening the Reporting of Observational Studies in Epidemiology (STROBE) guidelines for cross-sectional studies.

### Sleep assessment

Whole-night PSG (Alice 5LE, Philips Respironics, Eindhoven, the Netherlands) or whole-night PG (Alice NightOne, Philips Respironics, Eindhoven, the Netherlands; SOMNOcheck2, Weinmann, Germany) was performed in the SMC. All data were stored on a computer, manually scored, and evaluated according to the published American Academy of Sleep Medicine (AASM) and European Sleep Research Society (ESRS) guidelines by a certified sleep physician and technician^42^.

According to the results of whole-night PSG/PG, the severity of OSA was based on the AHI value, in accordance with the AASM diagnostic criteria and ESRS guidelines^42^. Following the whole-night polysomnography and/or polygraphy, we divided the total number of events (apnoeas and hypopneas) by the total number of hours the patient was asleep. Apnoea was defined as a complete cessation of air flow for 10 s or more, while hypopnea was defined as a decrease in air flow by more than 50% for 10 s or more, both followed by desaturation of 3% or more^42^. Therefore, according to the severity of OSA, the subjects were classified into groups: no OSA (AHI < 5; N = 17), mild to moderate OSA (AHI 5–29.9; N = 109), and severe OSA (AHI > 30; N = 79). Following the sleep assessment, subjects who consented to participate in this study were referred for saliva collection (N = 205) and periodontal examination (N = 142) (Fig. 1).

### Questionnaire

All of the subjects were interviewed by one examiner who was blinded to the PSG/PG reports. Data on medications (antidepressants, diuretics, antihypertensives, sedatives, bronchodilators, analgesics, antihistamines, anticonvulsants, antiparkinsonian drugs, anticholinergics, retinoids, anorexics, muscle relaxants, and decongestants) and concomitant diseases were collected from the patients’ medical records.

The questionnaire collected demographic data and an evaluation of the subjective assessment of dry mouth upon awakening with the responses on a Likert scale including never, rarely, sometimes, often, and almost always, as well as 5 more questions related to the subjective assessment of dry mouth during the daytime with yes/no answers and questions regarding oral hygiene habits^43,44^.

### Saliva assessment

#### Collection, pH measurement and storage

Unstimulated saliva was collected from all subjects between 9 and 12 am. The subjects were instructed not to eat food, drink beverages, smoke or chew gum 2 h prior to testing. During the test, subjects were advised to rinse out their mouth with water and relax for 1 min, swallow to void the mouth of saliva and then minimize movements for the next 15 min while collecting the saliva and gently spitting into the preweighted test tubes, as previously described by Navazesh and Kumar^4^. Immediately after collection, salivary pH was determined by the PICCOLO Plus pH tester with ± 0.01 accuracy and replaceable HI1295 16-cm (6.3") electrode with a temperature sensor (Hanna Instruments HI98113/Spectrum 240-73492, Smithfield, RI, United States). Prior to measuring the pH, the electrode was calibrated using standards at pH 4.0 and pH 7.0. After that, saliva samples were weighed with an analytical balance (KERN ALJ 220-4M). The weight of the preweighted empty test tubes was subtracted from the total weight of the saliva and the test tube to calculate only the mass of the collected saliva that was measured in g/min, which was considered equal to mL/min^4^. All saliva samples were frozen at − 18 °C and stored until biochemical analysis.

### Biochemical measurements

Calcium, phosphate and magnesium concentrations were measured at the Clinical Institute for Laboratory Diagnostics, Clinical Hospital Centre Zagreb with an Agilent 7500 cx (Agilent Technologies, Waldbronn, Germany) and inductively coupled plasma mass spectrometry (ICP‒MS). Saliva samples (400 μL) were digested with nitric acid (2 mL of 65% HNO_3_ and 1 mL of H_2_O) using high-pressure microwave digestion (UltraCLAVE, Milestone, Italy). After cooling, the samples were diluted with 1% (v/v) HNO_3_ to a total volume of 15 mL, and calcium, magnesium, and phosphates were analysed by ICP‒MS. All standard solutions were prepared from a 1-g/L PlasmaCAL standard (SCP Science, Canada). Seronorm® TraceElements Serum Control Level I and Level II (Sero AS, Billingstad, Norway) were used to control for the accuracy of the measurements. Free salivary cortisol was measured using a commercially available enzyme-linked immunosorbent assay (ELISA) produced by Demeditec Diagnostics GmbH, Kiel, Germany. Specifically, free cortisol from the tested saliva sample competed for binding with a cortisol-enzyme conjugate to a polyclonal antibody on a precoated microtiter well. The addition of a substrate caused colour development, which was measured spectrophotometrically at 450 nm and was inversely proportional to the concentration of free salivary cortisol in the saliva sample.

### Periodontal examination

Periodontal status was assessed immediately after saliva collection by two experienced periodontists (MR, PS). After examination of the same 10 participants, the achievement of an intraexaminer and extraexaminer reliability greater than 95% allowed the examiners to independently collect data. Out of 205 subjects, 63 were excluded from the periodontal examination: those who were edentulous or had fewer than two teeth (N = 13) and those who refused to participate in this study due to lack of time/interest (N = 50) (Fig. 1). The examination included assessment of the number of teeth, dental plaque, bleeding on probing and periodontal measurements: gingival recession (GR), PPD and CAL expressed in millimetres. GR was defined as the distance between the cement-enamel junction and the gingival margin, and the distance between the gingival margin and the bottom of the gingival sulcus was defined as the PPD. The sum of GR and the PPD was calculated as the CAL. These periodontal parameters were measured following previously published studies for periodontal assessment and diagnosis^45–47^. Based on those results, PISA scores were calculated using an online calculator available at www.parsprototo.info as previously described^48^.

### Statistics

The analysis was performed in SPSS (SPSS 14.0 Student Version for Windows) and MS Excel (Microsoft Corporation, 2018, Microsoft Excel). Data were tested for normality with the use of the Kolmogorov‒Smirnov test or the Shapiro‒Wilk normality test. Data are presented as the medians with interquartile ranges for continuous variables. Categorical variables are reported as frequencies and percentages for each investigated category. All reported differences were assessed with the use of the appropriate test for statistical significance. The names of all tests are described in the footnotes according to their use in the tables. When more than two comparisons were performed, the nonparametric Kruskal‒Wallis test was calculated for group differences, followed by the Mann‒Whitney test to assess the specific differences between groups. Categorical data reported as frequencies (percentages) were compared with the chi square test or Fisher’s exact test, depending on the variable.

Multiple linear regression was performed when salivary flow rate was included as a dependent variable, whereas independent variables included in the model were AHI (events/h), age, sex, BMI, salivary calcium, salivary phosphate, salivary magnesium, Ca/Mg, Ca/PO_4_, Mg/PO_4_ and salivary cortisol. Statistical significance was set at p < 0.05. The sample size was calculated following an analysis in MedCalc (MedCalc for Windows, version 19.1.2.) based on the correlation coefficient of the mean AHI and salivary flow rate (r = − 0.273) assessed in a pilot sample of 23 respondents, of whom 16 had a diagnosis of OSA (mild to severe) and 7 had no OSA. In the reported analysis, the α-level was 0.05, and power was set at 90%. The final sample size calculation was 136 respondents, which was increased towards the end of the study with the aim of having as many control subjects as possible.

## Supplementary Information

Supplementary Table 1.

Supplementary Table 2.

Supplementary Table 3.

## Data Availability

The data that support the findings of this study are available from the corresponding author, [KP], upon reasonable request.
