# Supplementary material for: Salivary parameters and periodontal inflammation in obstructive sleep apnoea patients
Source: Sci Rep. 2022 Nov 12;12:19387. doi: 10.1038/s41598-022-23957-5 (PMC9653442; doi:10.1038/s41598-022-23957-5)
Supplement: Supplementary file 1 — Supplementary Table 1. [file 41598_2022_23957_MOESM1_ESM.pdf]

Salivary parameters and periodontal inflammation in obstructive sleep apnoea patients

Mia Tranfić Duplančić[1], Renata Pecotić[1], Linda Lušić Kalcina[1], Ivana Pavlinac Dodig[1], Maja Valić[1], Marija Roguljić[1], Dunja Rogić[2], Ivana Lapić[2], Katarina Grdiša[2], Kristina Peroš[3*], Zoran Đogaš[1]

Supplementary Table 1. Medication intake (affecting hyposalivation) of the subjects according to OSA severity

		No OSA N=17	Mild to moderate OSA N=109	Severe OSA N=79	p
**Antidepressants**	No	16(94.1)	103(94.5)	77(98.7)	0.310
	Yes	1(5.9)	6(5.5)	1(1.3)	
**Diuretics**	No	17(100)	104(95.4)	77(98.7)	0.316
	Yes	0(0)	5(4.6)	1(1.3)	
**Antihypertensives**	No	14(82.4)	48(44)	34(43.6)	0.010
	Yes	3(17.6)	61(56)	44(56.4)	
**Sedatives**	No	17(100)	103(94.5)	75(96.2)	0.562
	Yes	0(0)	6(5.5)	3(3.8)	
**Bronchodilators**	No	17(100)	107(98.2)	77(98.7)	0.830
	Yes	0(0)	2(1.8)	1(1.3)	
**Analgesics**	No	16(94.1)	100(91.7)	73(93.6)	0.867
	Yes	1(5.9)	9(8.3)	5(6.4)	
**Antihistaminic**	No	16(94.1)	105(96.3)	78(100)	0.176
	Yes	1(5.9)	4(3.7)	0(0)	
**Anticonvulsants**	No	17(100)	108(99.1)	78(100)	0.645
	Yes	0(0)	1(0.9)	0(0)	
**Antiparkinsonian drugs**	No	17(100)	108(99.1)	78(100)	0.645
	Yes	0(0)	1(0.9)	0(0)	
**Other**	No	9(52.9)	54(49.5)	37(47.4)	0.907
	Yes	8(47.1)	55(50.5)	41(52.6)	
**Anticholinergics**	No	17(100)	109(100)	78(100)	NA
**Retinoids**	No	17(100)	109(100)	78(100)	NA
**Anorexics**	No	17(100)	109(100)	78(100)	NA
**Muscle relaxants**	No	17(100)	109(100)	78(100)	NA
**Decongestants**	No	17(100)	109(100)	78(100)	NA

Categorical data are presented as frequencies (percentages). Chi-square tests were used for comparisons among groups.
