# Supplementary material for: Salivary parameters and periodontal inflammation in obstructive sleep apnoea patients
Source: Sci Rep. 2022 Nov 12;12:19387. doi: 10.1038/s41598-022-23957-5 (PMC9653442; doi:10.1038/s41598-022-23957-5)
Supplement: Supplementary file 2 — Supplementary Table 2. [file 41598_2022_23957_MOESM2_ESM.pdf]

Salivary parameters and periodontal inflammation in obstructive sleep apnoea patients

Mia Tranfić Duplančić[1], Renata Pecotić[1], Linda Lušić Kalcina[1], Ivana Pavlinac Dodig[1], Maja Valić[1], Marija Roguljić[1], Dunja Rogić[2], Ivana Lapić[2], Katarina Grdiša[2], Kristina Peroš[3*], Zoran Đogaš[1]

Supplementary Table 2. Correlation of salivary parameters with AHI

	Total N=142	
	r	p
**Salivary flow**	0.05	0.532
**Salivary pH**	0.08	0.353
**Salivary calcium**	0.01	0.932
**Salivary phosphate**	-0.07	0.417
**Salivary magnesium**	0.07	0.44
**Ca/Mg**	-0.07	0.412
**Ca/PO_4**	0.02	0.782
**Mg/PO_4**	0.11	0.212
**Salivary cortisol**	-0.01	0.922
**Plaque**	0.26	0.003
**BoP**	0.06	0.487
**CAL**	0.13	0.132

Abbreviations: Correlation coefficient (r); Probability value (p)
