# Supplementary material for: Salivary parameters and periodontal inflammation in obstructive sleep apnoea patients
Source: Sci Rep. 2022 Nov 12;12:19387. doi: 10.1038/s41598-022-23957-5 (PMC9653442; doi:10.1038/s41598-022-23957-5)
Supplement: Supplementary file 3 — Supplementary Table 3. [file 41598_2022_23957_MOESM3_ESM.pdf]

Salivary parameters and periodontal inflammation in obstructive sleep apnoea patients

Mia Tranfić Duplančić[1], Renata Pecotić[1], Linda Lušić Kalcina[1], Ivana Pavlinac Dodig[1], Maja Valić[1], Marija Roguljić[1], Dunja Rogić[2], Ivana Lapić[2], Katarina Grdiša[2], Kristina Peroš[3*], Zoran Đogaš[1]

Supplementary Table 3. Linear regression analysis according to salivary flow rate

	B	SE	Beta	t	P	R^2	p
						32.9%	<0.001
**AHI**	0.001	0.001	0.063	0.746	0.457		
**Age**	-0.001	0.001	-0.055	-0.648	0.518		
**Sex (Male=0)**	-0.082	0.031	-0.201	-2.609	0.010		
**BMI**	0.001	0.003	0.031	0.344	0.732		
**Salivary calcium**	-0.040	0.114	-0.054	-0.350	0.727		
**Salivary phosphate**	-0.030	0.014	-0.408	-2.073	0.040		
**Salivary magnesium**	0.148	0.407	0.086	0.364	0.717		
**Ca/Mg**	0.026	0.022	0.203	1.188	0.237		
**Ca/PO_4**	-0.249	0.172	-0.139	-1.445	0.151		
**Mg/PO_4**	-4.641	2.753	-0.341	-1.686	0.095		
**Salivary cortisol**	-0.007	0.005	-0.115	-1.429	0.156		
**Hypertension**	0.007	0.032	0.019	0.229	0.820		
**DM II**	-0.003	0.047	-0.005	-0.056	0.955		
**Periodontal stage**	-0.033	0.022	-0.122	-1.534	0.128		

Abbreviations: Unstandardized beta (B); Standard error for the unstandardized beta (SE); Standardized beta (β); T-test statistic (t); Coefficient of determination (R^2); Probability value (p).
